# *Osmia* species (Hymenoptera, Megachilidae) from the southeastern United States with modified facial hairs: taxonomy, host plants, and conservation status

**DOI:** 10.3897/zookeys.148.1497

**Published:** 2011-11-21

**Authors:** Molly G. Rightmyer, Mark Deyrup, John S. Ascher, Terry Griswold

**Affiliations:** 1 USDA-ARS Bee Biology and Systematics Laboratory, BNR 244 UMC 5310, Utah State University, Logan, UT 84322-5310; 2 Archbold Biological Station, P.O. Box 2057, Lake Placid, FL 33862; 3 American Museum of Natural History, Division of Invertebrate Zoology, Central Park West at 79th Street, New York, NY 10024-5192

**Keywords:** Bee, Apoidea, Megachilinae, Osmiini, *Melanosmia*, *Osmia calaminthae*, *Osmia conjunctoides*, Lamiaceae, *Calamintha ashei*, oligolecty

## Abstract

We describe females and males of *Osmia (Melanosmia) calaminthae* **sp. n.**, an apparent floral specialist on *Calamintha ashei* (Lamiaceae), and provide observations on the behavior of female bees on flowers of this plant. We also provide diagnostic information for *Osmia (Diceratosmia) conjunctoides* Robertson, **stat. n.**, and synonymize *Osmia (Diceratosmia) subfasciata miamiensis* Mitchell with *Osmia conjunctoides* **syn. n.** Females of both *Osmia calaminthae* and *Osmia conjunctoides* are unique among North American *Osmia* for having short, erect, simple facial hairs, which are apparent adaptations for collecting pollen from nototribic flowers. *Osmia calaminthae* is currently only known from sandy scrub at four nearby sites in the southern Lake Wales Ridge in Highlands County, Florida, USA, while *Osmia conjunctoides* is known from limited but widespread sites in the southeastern USA. We discuss the conservation status of both species based on known or speculated floral associates and distributions.

## Introduction

The genus *Osmia* in North America comprises about 150 described species (Ascher and Pickering 2011) that are usually metallic green or blue, sometimes brilliantly so. The 30 species recorded east of the Mississippi River prior to this contribution have been relatively well studied taxonomically (Sandhouse 1939, Mitchell 1962), and the internet houses freely available matrix-based and dichotomous keys to the species (Arduser 2009, Griswold et al. 2010, Andrus et al. 2010). As in many other bee genera, the species diversity in eastern North America is depauperate compared to west of the Mississippi River. Nonetheless, there remain many areas in eastern North America that would benefit from increased study and collection effort. In particular, Florida and other states in the southeastern United States appear to house a number of interesting bee endemics, and new state records continue to be documented from this region (Hall and Ascher 2010).

**Figures 1–3.**
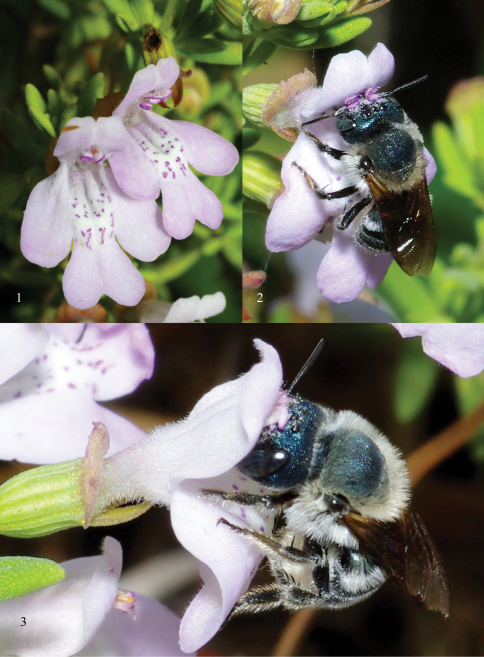
**1** Flowers of *Calamintha ashei* (Weath.) Shinners (Lamiaceae) **2–3** *Osmia calaminthae*, sp. n., visiting flowers of *Calamintha ashei* at Lake Placid, Highlands County, Florida. Photographs by T. Lethbridge.

**Figure 4.**
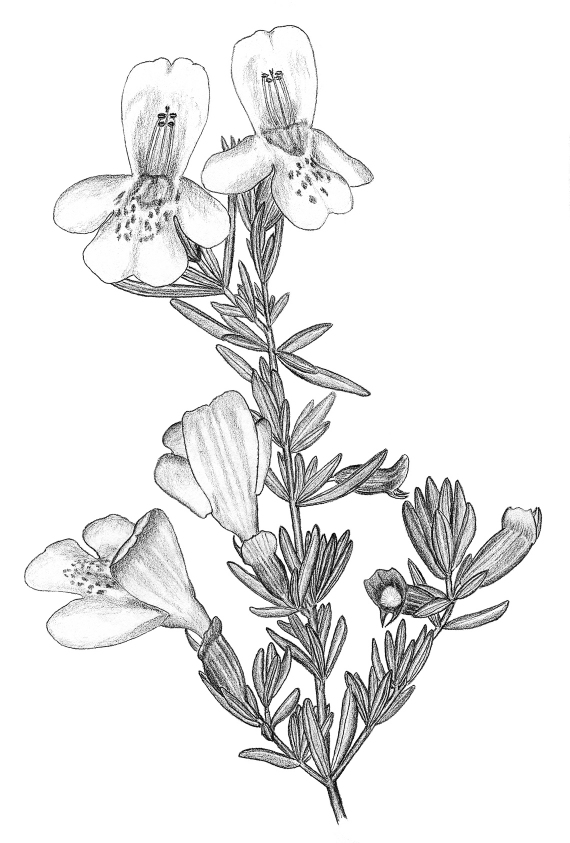
Habitus illustration of *Calamintha ashei*, the only known floral host of *Osmia calaminthae* sp. n. Illustration by M. Deyrup.

Herein we describe one such find, *Osmia (Melanosmia) calaminthae* sp. n., known only from Highlands County, Florida, in sandy scrub at the southern end of the Lake Wales Ridge. This habitat houses many Florida endemic plants and animals, including bees (Deyrup et al. 2002). *Osmia calaminthae* is apparently a floral specialist on *Calamintha ashei* (Weath.) Shinners (Lamiaceae), Ashe’s Calamint (Figs 1, 4; also known as Ashe’s Savory), a threatened woody mint found in sand pine/scrub habitat in the Florida central highlands and southeastern Georgia (Coile 2000). The new species was discovered by Deyrup et al. (2002) during surveys of the Archbold Biological Station. During subsequent searches by J. S. Ascher, H. G. Hall, and colleagues, including photographer T. Lethbridge, numerous females and a few males were found visiting the host plant in late morning at the Placid Lakes Development southwest of the town of Lake Placid (N27.250, W81.389). At this site the host plant grows commonly in sand scrub, much of which occurs in vacant lots within a partially completed housing subdivision.

**Figures 5–8.**
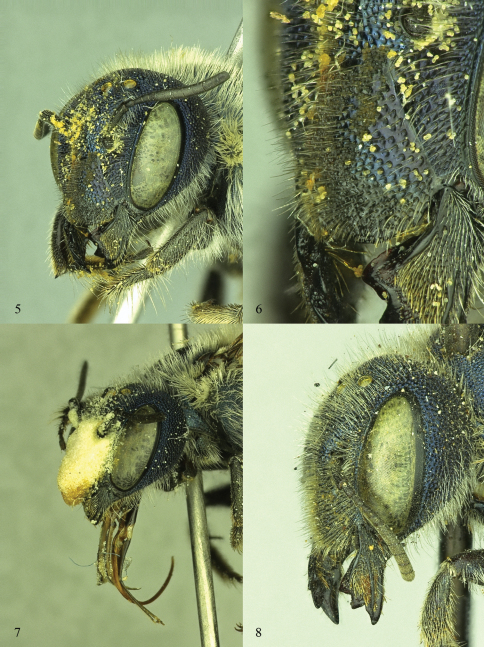
Oblique view of female *Osmia* heads **5, 6** *Osmia calaminthae*, holotype specimen **6** Close up of clypeus and paraocular area **7** *Osmia calaminthae*, paratype specimen, showing pollen mass on face **8** *Osmia conjunctoides* (holotype specimen of *Osmia subfasciata miamiensis*).

*Osmia calaminthae* females have modified hairs on the face that readily distinguish the species from similar species of *Osmia* (see Diagnosis, below). Modified facial hairs for collecting pollen from nototribic flowers occur rarely in scattered species across multiple bee families (e.g., Müller 1996, Ayala and Griswold 2005, Michener 2007). In *Osmia calaminthae*, the hairs on the frons, clypeus, and scape are uniformly short, erect, and simple, and apparently function to trap pollen when the female enters the corolla and her head contacts the anthers of this flower (Figs 2, 3, 5–7). The pollen accumulates to form a conspicuous mass and evidently remains on the face of the bee for an extended time during foraging bouts (as opposed to being immediately transferred to the metasomal scopa), as evidenced by conspicuous loads adhering to the face of approximately one-fourth of the female specimens examined. Interestingly, another *Osmia* that is also found in the southeastern United States, *Osmia (Diceratosmia) conjunctoides* Robertson, new status, has nearly identical hairs on the frons and clypeus of females (Fig. 8). *Osmia (Diceratosmia) subfasciata miamiensis* Mitchell, 1962, was described as a subspecies but is herein distinguished from typical *Osmia subfasciata subfasciata* Cresson and newly synonymized under *Osmia conjunctoides*. Specimens and floral records are scarce for *Osmia conjunctoides* (Pascarella 2008); only one examined specimen has an associated floral record, *Crotalaria pumila* Ortega (Fabaceae), which limits our interpretation of floral associations for this bee.

It is the honor of MGR and JSA to dedicate this paper to Kumar Krishna in celebration of his lifelong achievements in the study of Isoptera. Both authors are grateful to Kumar and Valerie Krishna for the kindness and generosity shown to them during their respective tenures at the American Museum of Natural History.

## Methods

The morphological terminology follows that proposed by Michener (2007) and Harris (1979). Mandibular teeth are numbered from ventral-most tooth to dorsal-most tooth. Thus, the ventral-most tooth is the first tooth and the next ventral-most tooth is the second. In the species treated herein, between the second and dorsal-most tooth is a smaller, slightly more interior, cutting edge extended from the dorsal-most tooth, here called the third tooth. The dorsal-most tooth is the fourth tooth.

The following morphological abbreviations are used: flagellar segment (F), metasomal tergum (T), metasomal sternum (S), and ocellar diameter (OD). Measurements are given for the holotype specimen, with the observed range from other specimens following in parentheses.

Bee specimens were examined and measured using a Leica MZ12 dissection microscope and ocular micrometer. Pollen grains were slide mounted in silicone oil and examined using a Nikon E200 compound microscope. Photomicrographs of pinned specimens were taken using a Keyence VHX-500F Digital Imaging System.

The following abbreviations are used for specimen repositories, with individuals associated with those repositories following in parentheses:

**Champaign** – Illinois Natural History Survey, Champaign IL (D. Dmitriev)

**New York** – American Museum of Natural History, New York NY (J. S. Ascher, J. G. Rozen, Jr.)

**Gainesville** – Florida State Collection of Arthropods, Gainesville FL (J. Wiley)

**Lake Placid** – Archbold Biological Station, Lake Placid FL (M. Deyrup)

**Logan** – USDA-ARS Bee Biology and Systematics Laboratory, Logan UT (T. Griswold, H. Ikerd)

**Orlando** – University of Central Florida, Orlando FL (S. M. Fullerton, S. Kelly)

**Raleigh** – North Carolina State University, Raleigh NC (R. Blinn)

**Washington, D.C.** – United States National Museum of Natural History, Washington D.C. (S. G. Brady, B. Harris)

## Taxonomy

### Osmia (Melanosmia) calaminthae

Rightmyer, Ascher & Griswold sp. n.

urn:lsid:zoobank.org:act:DB373E46-E362-4108-8AD5-52DE1598A8FD

http://species-id.net/wiki/Osmia_calaminthae

Figs 2, 3 5–7 9 [Fig F5] 26

Osmia sp.; Deyrup et al. 2002: 99.

#### Diagnosis.

**Figures 9–14.**
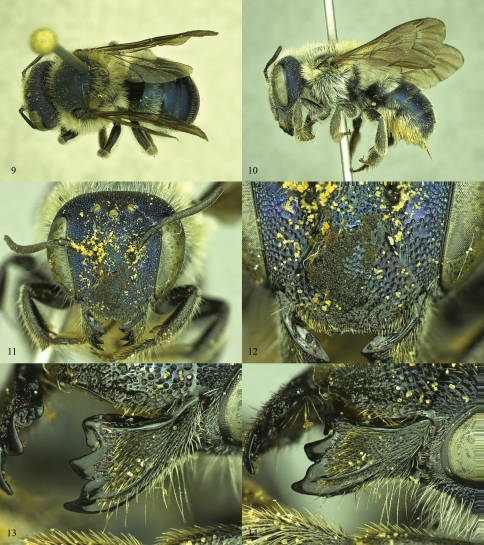
*Osmia calaminthae*, holotype female **9** Dorsal habitus **10** Lateral habitus **11** Face **12** Close up of clypeus and paraocular area **13** Mandible, showing the shape and placement of teeth **14** Mandible, showing outer and condylar ridges and overall shape.

**Figures 15–20. F5:**
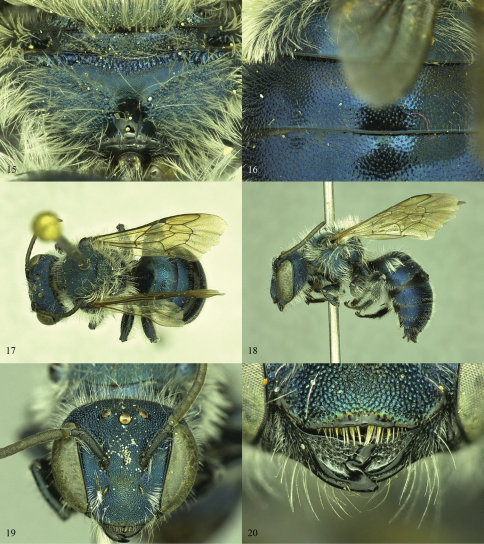
**15, 16** *Osmia calaminthae*, females **15** Propodeal triangle of paratype specimen **16** T1–T3 of holotype specimen **17–20** *Osmia calaminthae*, male paratype **17** Dorsal habitus **18** Lateral habitus **19** Face **20** Mandibles

Females of *Osmia calaminthae* are most similar to *Osmia (Melanosmia) albiventris* Cresson and *Osmia (Melanosmia) cordata* Robertson, sharing with those species the white hairs of the body (including scopa) and four-toothed mandible with the outer and condylar ridges parallel (Figs 13, 14). Unlike those species, *Osmia calaminthae* has specialized hairs on the face (including clypeus and frons) that are extremely short, evenly spaced, simple, and stout (Figs 5, 6; longer and finer in *Osmia albiventris* and *Osmia cordata*). In addition, the punctures of the head and mesosoma are large and deeply impressed in *Osmia calaminthae*, the hairs on the posterior surface of the foretarsal segments are relatively long, the wings are heavily infuscate, the rugose sculpturing of the dorsal propodeal triangle is strongly impressed and well differentiated from the ventral area of shagreened integument (Fig. 15), and the hairs on the lateral dorsal surface of T1 are dense and long, much more so than on remaining metasomal terga (Figs 9, 10; T1 hairs not conspicuously longer and denser than those on other metasomal terga in *Osmia albiventris* and *Osmia cordata*). *Osmia (Melanosmia) sandhouseae* Mitchell is a superficially similar species found sympatrically in Florida and is known to visit *Calamintha* in March (Deyrup et al. 2002); however, in that species the outer and condylar ridges of the mandible converge apically (parallel in *Osmia calaminthae*) and the hairs of the clypeus and frons are long and fine.

Males of *Osmia calaminthae* are distinguished from many other *Osmia* by the relatively slender, pointed teeth of T7 (basally about one-fourth the width of the midapical emargination; Fig. 22). Among the *Osmia* with such slender teeth on T7, *Osmia calaminthae* is extremely similar to *Osmia cordata* due to the white hairs on the metasomal terga (including laterally on T6) and S4 (Fig. 24), and by the hairs on the apical margin of S4 that are longer medially than laterally. *Osmia calaminthae* can be separated from *Osmia cordata* by the deep and large punctures on the frons and vertex. Males of *Osmia sandhouseae* Mitchell are superficially similar; however, in that species the punctures on the upper gena are extremely large and deep, much more so than on the vertex, while in *Osmia calaminthae* the punctures of the upper gena and vertex are subequal in size.

**Figures 21–26.**
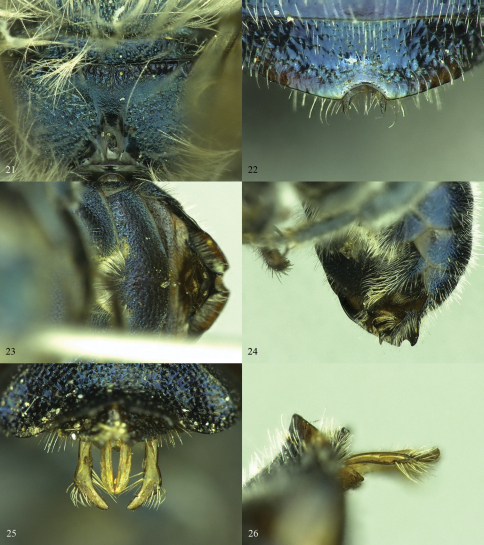
*Osmia calaminthae*, male paratypes **21** Propodeal triangle **22** T6 and T7 **23** S3 and S4, dorsal view **24** S3 and S4, oblique view **25** Genital capsule, dorsal view **26** Genital capsule, lateral view.

#### Description.

**Female.** Figs 5–7, 9–16. Total length: ca. 11 mm (10–11 mm); Forewing length: 7 mm (6–7 mm); distance between lateral ocellus and preoccipital margin 0.6 mm (0.5–0.6 mm); distance of lateral ocellus to compound eye 0.6 mm.

*Color*: Dark blue (mesosoma sometimes with integument slightly paler blue), except with brown integument on mouthparts, labrum, mandible, apical edge of clypeus, antenna, legs distal to trochanters, apical margins of metasomal terga, and metasomal sterna. Wings strongly infuscate.

*Pubescence*: Hairs entirely white to pale golden except darker golden on mouthparts and distally on legs, brown on wings; hairs conspicuous on mesosoma and T1 so as to obscure underlying blue integument, but inconspicuous on distal terga. Galea and basal two labial palpal segments with hairs on lateral margins straight, 0.2–0.5 OD in length. Labrum with long hairs arranged in two curved, transverse rows, along subapical margin and at approximate midpoint (hairs slightly more scattered along row at midpoint), with additional fringe of shorter hairs at apical margin. Clypeus below apical margin with lateral tuft of pale golden, medially directed hairs (often hidden by clypeal margin). Head with short, stout, simple, erect hairs evenly spaced on face (Figs 5, 6), scape, and pedicel, these hairs denser and appressed on outer surface of mandible, longer and relatively sparse on ventral margin of mandible, vertex, and posteriorly on gena. Hypostomal area with straight, minutely branched hairs evenly distributed across area, 2.0–3.0 OD in length. Mesosoma (excluding legs and wings) and laterally on T1 covered with dense, long, minutely branched, white hairs (Figs 9, 10); remainder of metasomal terga with hairs conspicuously shorter and sparser than hairs on mesosoma and T1. Legs with hairs on outer surfaces white, on forefemur and foretibia relatively slender and minutely branched, on foretarsal segments long (ca. 3.0 OD in length), slightly stouter, and simple; on midleg outer surface entirely minutely branched; on hindleg outer surface mostly minutely branched except basitarsus with hairs appressed, simple, and relatively stout. Legs with hairs on inner surfaces of fore- and midfemora and fore- and midtibiae white, minutely branched, and relatively sparse, with some shining areas lacking hairs; inner surface of hindfemur with denser, minutely branched white hairs, of hind tibia with very dense, short, white, subappressed hairs; inner surfaces of all tarsal segments with hairs dense, golden, stout, and simple. Wing membranes with short, dense, evenly distributed, simple hairs. Scopa white to pale golden.

*Punctation*: Head and mesosoma with punctures nearly contiguous, round, and strongly impressed except as follows: labrum mostly impunctate; clypeus with impunctate midapical truncation about half length of F1; clypeus immediately adjacent to apical impunctate truncation and next to compound eye with punctures relatively small and dense (Fig. 12); paraocular area with punctures separated by up to two puncture diameters; clypeus, vertex, and mesoscutum immediately posterior to median longitudinal sulcus with punctures separated by up to one puncture diameter; mesepisternum in upper anterior corner with punctures relatively small, ventrally with punctures separated by up to one puncture diameter; hypostomal area, pronotum, propodeum, and legs with punctures shallowly impressed, sometimes elongated into oval shape; tegula with punctures minute, dense at margins and sparse medially (separated by up to four or five puncture diameters); metanotum, metepisternum, and lateral and posterior surfaces of propodeum with background integument moderately granulose and relatively dull; propodeal triangle with dorsal third strongly, deeply areolate to lineate, lower two thirds granulose grading to shining, glabrous area along lower lateral margin (Fig. 15). T1 anterior and dorsal surfaces, and T2–T3 shining, T4–T6 moderately shagreened, T1 medially on disc with small punctures separated by up to a puncture diameter, grading to slightly larger and denser punctures on more posterior terga, T1–T5 apical impunctate bands relatively narrow laterally, ca. one puncture diameter in length, medially with impunctate bands widened, up to four puncture diameters in length (or even longer at exact midpoint).

*Structure*: Labial palpus four-segmented, second labial palpal segment ca. one-fourth longer than basal-most segment. Mandible with outer and condylar ridges of subequal thickness or with condylar ridge slightly thicker, parallel along length (Fig. 14); apical margin with four teeth, third separated from second and fourth by carina, margin of third tooth forming distinct V-shape with adjacent margin of second and weakly curved U-shape with adjacent margin of fourth, third tooth weakly set back from second and fourth (Fig. 13); inner, ventral margin of mandible lacking distinct tooth, very weakly diverging away from condylar ridge basally; mandible apically widened (1.5 times wider than median width), first tooth length subequal to that of other teeth or very slightly longer, second tooth located about half way between first and fourth teeth (Fig. 13). Clypeus with apical margin forming anteriorly produced truncation, linear or weakly concave along truncation and forming ca. 130 degree angle with lateral apical margin of clypeus. F1 one-third longer than F2 length or slightly more, remaining apical flagellar segments gradually increasing in length such that F10 ca. one-fourth longer than F1 length. Vertex behind lateral ocellus 2.5–3.0 OD in length. Genal width subequal to that of compound eye in lateral view. Preoccipital margin rounded, not carinate. Hypostomal carina moderately high, highest at about midpoint of hypostomal area posterior to angle, not forming triangular projection at this point but forming distinct, semicircular projection, tapering to low carina or near obsolescence at angle. Malus forming pointed apical spine, this spine more or less a narrowed continuation of nearby edge of velum. Foretarsal segments excluding basitarsal and apical-most segments with lobes moderately swollen, anterior lobes slightly longer than posterior. Midtarsal segments with anterior and posterior lobes of equal width, weakly swollen; hind tarsal segments not swollen. Hind tibial spurs more or less straight on basal three-fourths, with outer spur moderately curved at apical tip and inner spur slightly less strongly curved apically, outer spur about a fifth to a sixth shorter than inner. Hind basitarsus with lateral margins of outer surface parallel.

**Male.** Figs 17–26. Total length: ca. 10 mm; Forewing length: 6 mm; length of lateral ocellus to preoccipital margin 0.4 mm; length of lateral ocellus to compound eye 0.5 mm.

*Color*: Head and mesosoma pale blue, metasoma dark blue, except with brown integument on mouthparts, labrum, mandible, apical edge of clypeus, antenna, legs distal to trochanters, S5–S8, and apical margins of all metasomal terga and S1–S4. Wings moderately infuscate, except along leading edge of forewing more strongly infuscate.

*Pubescence*: White, minutely branched hairs on body except golden to pale golden, stouter hairs on inner surfaces of tarsi. Labrum sparsely covered with hairs on apical half and with hairs forming short fringe at apical margin. S2 with hairs at apical third relatively long (ca. 3.0 OD). S3 with dense, posteriorly directed hairs forming semicircular fringe along entire emargination (hairs ca. 1.5 OD in length throughout) (Fig. 23). S4 sparsely covered with white, medio-posteriorly directed, distally wavy hairs, these hairs not interrupted medially on S4, distinctly longer at midapical truncation than laterally on apical margin of disc. S6 midapical truncation sparsely covered with short, white hairs.

*Punctation*: Head with punctures ovate to circular, contiguous or nearly so and deeply impressed except as follows: labrum mostly impunctate on basal half; clypeus with impunctate area immediately next to anterior tentorial pit and impunctate band along apical margin about one-fourth length of F1 and slightly swollen on median third (Fig. 20); disc of clypeus and interantennal area with punctures small and ovate; hypostomal area anteriorly near angle with punctures weakly, shallowly impressed. Mesosoma with punctures round, nearly contiguous and deeply impressed except as follows: tegula with punctures minute, sparser medially, separated by up to eight puncture diameters; metepisternum with punctures more irregular and with impunctate area near anterior margin and sometimes medially across sclerite; pronotum and lateral and posterior surfaces of propodeum strongly shagreened, with very weakly, shallowly impressed punctures; metanotum with punctures distinct but smaller than on mesoscutum and separated by about a puncture diameter; propodeal triangle strongly lineolate to reticulate on dorsal half and shagreened on lower half, sometimes with weakly shining areas laterally near ventral margin (Fig. 21); legs with inner surfaces of trochanters, femora, and tibiae (except hind tibia) shining, with scattered smaller punctures. T1 with anterior surface weakly shagreened, shining; metasomal terga with dorsal surfaces very weakly shagreened, shining. Metasomal terga with punctures small and well impressed (slightly less impressed on T5–T7). T1–T4 dorsal surfaces with punctures separated between 0.5 and 2.0 puncture diameters; apical impunctate margins medially ca. 3.0–4.0 puncture diameters in length, laterally as little as 1.0 puncture diameter. T5–T6 with punctures less distinct, separated by ca. 1.0 puncture diameter medially; T5 with apical impunctate margin medially ca. 3.0 puncture diameters in length. S1–S3 with punctures moderately impressed, ovate. S4 with integument punctate basally, grading to shagreened and papillate at bases of hairs apically. S5–S6 shagreened.

*Structure*: Mandible with outer and condylar ridges converging apically; with two teeth, upper and lower teeth nearly the same width and length; inner margins of upper and lower teeth forming nearly 90 degree angle; upper tooth with inner and dorsal margins forming ca. 45–60 degree angle; inner, ventral margin of mandible weakly diverging away from condylar ridge basally. Clypeus apical margin lacking distinct apical truncation, medially very weakly concave, laterally with weakly tuberculate swelling. Flagellar segments subequal in length, except F1 about three-fourths length of F2 and F11 slightly longer than other segments. Vertex behind lateral ocellus 1.5 OD in length or slightly longer. Genal width ca. three-fourths that of compound eye in lateral view or slightly wider. Preoccipital margin rounded, not carinate. Hypostomal carina relatively shallow, about the same height along length of head, gradually tapering to near obsolescence at angle, not forming distinct tooth. Malus forming small but distinct, pointed apical spine. Foretarsal segments excluding basitarsal and apical-most segments with anterior lobes very slightly more swollen than posterior. Mid- and hind tarsal segments not swollen. Hind tibial spurs relatively stout, very weakly curved along length, outer spur slightly shorter than inner. Hind basitarsus with lateral margins of outer surface subparallel, with very small tooth on inner margin about one-fourth from apical margin along length. T6 midapically with moderate emargination, forming one-half of circle in outline or nearly so (Fig. 22); T6 lateroapical margin smoothly, weakly convex, not forming distinct tooth or forming very weak lateral tooth. T7 midapically strongly emarginate, forming semicircle about as wide as deep (slightly smaller than 1.0 OD in width), with spines on either side of emargination slender, about one-fourth as wide as emargination width (Fig. 22). S2 evenly convex, covering most of S3 (in one specimen with weak emargination at midapex). S3 with midapical emargination strongly semicircular, about as wide and long (half entire width of sternum, 1.5 OD in length or slightly more, measuring only apical margin of sternum and not including basal fringe of hairs; Fig. 23). S4 midapically with wide, poorly defined truncation (about third width of entire sternum). S6 with midapical truncation ca. one-fifth width of sternum, truncation as wide as long, apical margin of truncation distinctly emarginate. Gonoforceps narrowed apical to subapical bend, weakly pointed at apical tip in dorsal view (Fig. 25), more or less straight along length in lateral view (Fig. 26).

#### Distribution.

 Known only from Highlands County, Florida.

#### Holotype female.

“USA: FL [Florida]: Highlands Co. Lake Placid, 18 March 2002, J. S. Ascher, ex: *Calamintha ashei*//HOLOTYPE ♀ *Osmia calaminthae* Rightmyer, Ascher, Griswold [red label]” (New York). The type locality is southwest of the town of Lake Placid in an area of the subdivision of Placid Lakes Development that still includes many vacant lots: N27.2502, W81.3898.

#### Paratypes.

**USA**: **FLORIDA**, **Highlands Co.**, Lake Placid, 18 March 2002, *Calamintha ashei*, J. S. Ascher (4♀, New York; 1♀, Logan), Archbold Biological Station, 28 March 1988, *Satureja ashei*, A. Warneke (1♀, Lake Placid), 29 March 2000, *Calamintha ashei*, M. Deyrup (1♀, Lake Placid), 10 April 2001, *Calamintha ashei*, M. Deyrup (1♀, Lake Placid), 18 April 1983, H. L. Dozier (1♀, Gainesville), 25 April 1983, *Lupinus diffusus*, A. Schreffler (1♀, Gainesville), Archbold Biological Station, Junction roads 40 & 36 Rosemary Bald, 27 March 2001, *Calamintha ashei*, M. Deyrup (2♀, Lake Placid); Lake Wales Ridge Wildlife and Environmental Area, Gould Road Preserve, N27.13657, W08132495, 15 March 2009, *Ceratiola* scrub, Townes and bowl traps, M. & N. Deyrup, A. May, H. Otte (4♀, Lake Placid; 1♀, Logan); Lake Wales Ridge Wildlife and Environmental Area, Holmes Avenue Preserve, N27.28097 W081.31862, 24 April 2009, *Calamintha ashei*, M. Deyrup (1♀, Lake Placid); Placid Lakes, 8 April 2001, *Calamintha ashei*, Florida scrub habitat, M. Deyrup (3♀, Lake Placid); Placid Lakes Development, 16 March 2002, *Calamintha ashei*, sand pine scrub habitat, M. Deyrup (3♀, Gainesville; 4♀, Lake Placid; 1♀, Logan), 29 March 2007, *Calamintha ashei*, M. Deyrup (2♀, Lake Placid), 30 March 2009, *Calamintha ashei*, J. S. Ascher, D. Webber (3♀, 2♂, New York), 31 March 2009, *Calamintha ashei*, J. S. Ascher, D. Webber (8♀, New York), 3 April 2006, *Calamintha ashei*, J. S. Ascher, C. Dong (4♀, New York), 4 April 2006 (8♀, New York; 1♀, Logan).

#### Additional records.

Four females were collected by H. G. Hall at the type locality on 31 March and others were observed and photographed there by T. Lethbridge on 31 March and 2 Apr 2010 (Figs 1–3; additional photos and informative captions here: http://bugguide.net/node/view/394002/bgimage).

#### Etymology.

 The name “calaminthae” is Latin, referring to mint, and is derived from the name of its presumed pollen host plant.

### Osmia (Diceratosmia) conjunctoides

Robertson stat. n.

http://species-id.net/wiki/Osmia_conjunctoides

Figs 8 27 32

Osmia conjunctoides Robertson 1893: 276; Sandhouse 1939: 140 [synonymy with *Osmia subfasciata*]; Mitchell, 1962:83 [synonymy with *Osmia subfasciata subfasciata*]; LaBerge (in Webb 1980: 118) [lectotype designation].
Diceratosmia subfasciata conjunctoides (Robertson); Michener 1949: 264 [diagnosis].
Osmia (Diceratosmia) subfasciata miamiensis Mitchell 1962: 84. syn. n.

#### Diagnosis.

Females of this species are distinguished from all other *Diceratosmia*, including typical *Osmia (Diceratosmia) subfasciata*, by the nearly uniformly short, straight to slightly hooked hairs on the clypeus and slightly longer hairs on the frons (Fig. 8). *Osmia conjunctoides* is also distinguished from *Osmia subfasciata* by the scopal hairs: in *Osmia conjunctoides*, the apical tips of the hairs on S2 and S3 are weakly tapered, while in *Osmia subfasciata* the hairs are blunt, widened and slightly rounded at their apical tips. The form of clypeal hairs in the female is very similar to that of *Osmia (Melanosmia) calaminthae*; however, in that species the punctures of the metasomal terga are not so large (compare Figs 16 and 32), there is no carinate ridge on the hind coxa, the parapsidal line is punctiform, and the metasomal terga (especially T1 and T2) lack the distinct, short, dense, pale, apicolateral hair bands characteristic of subgenus *Diceratosmia* (in *Osmia calaminthae* T1 has dense, pale hairs, but these hairs are long and contrast with the short, sparse hairs on T2).

Males of *Osmia conjunctoides* are extremely similar to *Osmia (Diceratosmia) subfasciata*. Finding reliable characters to distinguish the two species is made problematic by the availability of only seven male specimens of *Osmia conjunctoides*. This material suffices to permit the two species to be differentiated by the following characters: In *Osmia conjunctoides*, the mesoscutum is more finely and densely punctate than in *Osmia subfasciata* (*Osmia conjunctoides* with ca. 16 punctures between parapsidal line and midline, these punctures distinctly smaller than those on the scutellum; *Osmia subfasciata* with ca. 11 punctures between parapsidal line and midline, these punctures about the same size as those on the scutellum). In dorsal view, T1 of *Osmia conjunctoides* is less concave along its anterior margin, while in *Osmia subfasciata* the anterior margin is strongly curved, forming anterolaterally rounded corners. In addition, *Osmia conjunctoides* is usually a slightly larger bee than *Osmia subfasciata* (6–7 mm vs. 8–9 mm); all examined *Osmia conjunctoides* from Florida and Georgia are dark blue, while all examined *Osmia subfasciata* from throughout its range are a paler greenish blue; however, the male specimen of *Osmia conjunctoides* from Mississippi is greenish blue, similar to *Osmia subfasciata*. In *Osmia conjunctoides*, the lower propodeal triangle tends to be weakly shagreened throughout, while in *Osmia subfasciata* the lower propodeal triangle tends to be shining. In addition, T6 of *Osmia conjunctoides* has an apical, upturned flange that is longer than in examined specimens of *Osmia subfasciata* (ca. 2.0 adjacent puncture diameters in the former versus 1.0 adjacent puncture diameters in the latter).

**Figures 27–32.**
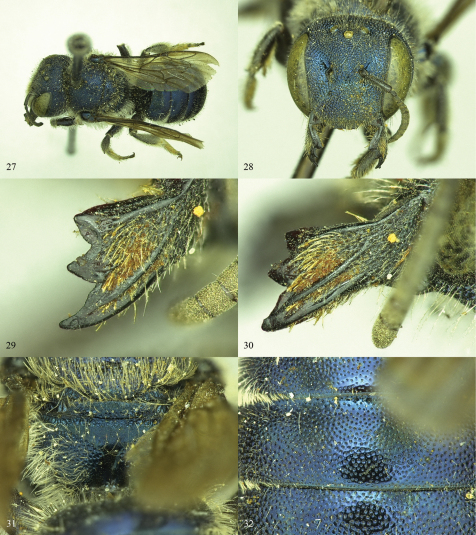
*Osmia conjunctoides* (female holotype of *Osmia subfasciata miamiensis*) **27** Dorsal view. **28** Face **29** Mandible, showing the shape and placement of teeth **30** Mandible, showing outer and condylar ridges and overall shape **31** Propodeal triangle **32** T1–T3.

#### Material examined.

**USA: FLORIDA, Citrus Co.**, Inverness, Robertson [1♂, New York], 17 February 1891 [1♂, Champaign (holotype of *Osmia conjunctoides*)]; **Highlands Co.**, Highlands Hammock State Park, 14 April 1968, malaise trap, H. V. Weems Jr. [1♀, Gainesville]; **Hillsborough Co.**, Lutz, 17 March 1926, Krautwurm [1♀, Logan]; **Liberty Co.**, Torreya Ravine, 15 April 1938, F. E. Lutz (1♀, New York); **Miami-Dade Co.**, Cape Florida, 15 February 1925, *Crotolaria*, S. Graenicher [1♀, Washington DC (holotype of *Osmia subfasciata miamiensis*)]; Miami Beach, 8 February 1917, Graenicher [1♂, Raleigh (allotype of *Osmia subfasciata miamiensis*)]; **Seminole Co.**, Lower Wekiva River Preserve State Park, Burn Zone LW-10, S39 T19S R29E, LLP-Turkey Oak, P. Russell, S. Fullerton, 6 February 2001, blue pan trap (1♂, Orlando), 19 February 2001, yellow pan trap (1♂, Orlando), blue pan trap (1♂, Logan); **GEORGIA, St. Catherines Island**, 16–22 April 1983, Rozen, Favreau, Stupakoff (1♀, New York); **MISSISSIPPI, Forrest Co.**, Hattiesburg, 12 March 1944, C. D. Michener (1♂, New York), 6 April 1944 (1♀, New York).

#### Comments.

Graenicher (1930) provides brief collecting notes on this species, under the name *Osmia subfasciata*:“This species occurs in the sand dunes at Miami Beach and on Biscayne Key (across the Bay southeast of Miami), and visits the flowers of *Crotalaria pumila*. Dates of capture: February 8, 15, and March 17.”

Michener (1949: 264) considered a male specimen of *Osmia conjunctoides* from northern peninsular Florida to intergrade with the typical *Osmia subfasciata* in features of T6 and T7 (although not in color). However, he was apparently unaware of the distinct facial and scopal hair features distinguishing females of *Osmia conjunctoides* from *Osmia subfasciata*. Specimens of *Osmia conjunctoides* that we have examined from northern peninsular Florida as well as Mississippi and Georgia are consistent with those from southern Florida in these diagnostic features of the females as well as in the finer punctures of the male mesoscutum. Although *Osmia conjunctoides* and *Osmia subfasciata* *sensu stricto* are extremely similar in the male S4, we do not agree with the historic placements of this bee as a subspecies or synonym of *Osmia subfasciata* due to the consistent differences in male punctation and female clypeal and scopal hairs.

According to Mitchell (1962: 84), two additional female paratype specimens of *Osmia subfasciata miamiensis* exist in Raleigh with the same label data as the male allotype. Pascarella (2008) also recorded the species from “Charlotte Harbor,” possibly based on distributional records found in Michener (1949), but we have not been able to confirm this record. Until we have access to further material, *Osmia conjunctoides* is provisionally considered to range from southern Florida north to Georgia and Mississippi, while we can confirm the presence of typical *Osmia subfasciata* from northeastern Mexico and southeastern California southeast to Alabama and South Carolina, and northeast to Illinois and New Jersey.

## Natural history and conservation status

### Osmia calaminthae

As indicated by its specific epithet, *Osmia calaminthae* appears to be closely associated with the woody mint *Calamintha ashei* (Figs 1, 4). Individual plants of this species may persist for at least a decade, possibly considerably longer. The flowering period is primarily from mid-March through mid-April; there can be variation in flowering phenology within this main blooming period among individuals growing in close proximity. Individual mature plants present hundreds of flowers over a period of several weeks. For flower visitors, *Calamintha ashei* provides a dependable annual resource, even when the population of mature plants is small; as few as 20–30 plants may produce thousands of flowers each season.

Flowers have a corolla about 7 mm long, strongly protected against nectar-robbers by a stiff calyx that subsequently functions as a seed capsule (Figs 1, 4). There is almost no evidence of nectar robbing. Pollen is produced by four anthers whose filaments lie along the fused upper petals. Anthers are arranged in two rows, approximately marking the corners of a square (Fig. 1). In late morning and early afternoon when the anthers open, visiting female *Osmia calaminthae* rapidly bob their heads three to four times upon entering a flower (Figs 2, 3). This behavior, unusual in bees, might be associated with dislodging pollen, although this remains to be investigated. Pollen grains scraped from the head of one paratype specimen of *Osmia calaminthae* were found to be consistent with those described for *Calamintha*: three ridges were observed in lateral view, suggesting a hexacolpate condition (Trudel and Morton 1991). The behavior exhibited by *Osmia calaminthae* has not been observed in 23 other species of bees that visit flowers of *Calamintha ashei* (Deyrup et al. 2002). Approximately one-fourth of the examined females had a large quantity of pollen trapped on the clypeus, supraclypeal area, frons, and scape (i.e., with pollen masses conspicuously visible to the naked eye, similar to Fig. 7). The large pollen load on the face suggests that females of *Osmia calaminthae* do not immediately groom the pollen back to the scopa when foraging between flowers. Such large pollen masses may even indicate that females are able to use the modified hairs on the face, in addition to the metasomal scopa which also contained pollen, to transport pollen to the nest; however, this was not directly observed and should be investigated in the future.

Flowers of *Calamintha ashei* are also visited by flies in the family Bombyliidae: *Bombylius* spp., *Systoechus solitus* (Walker), *Geron* sp. and *Toxophora* sp., as well as a member of the Syrphidae: *Copestylum florida* (Hull). The occurrence of numerous alternative potential pollinators adds some resilience to a pollination system that includes a rare plant and an apparently specialized bee. The only species of visitor that occurs in large enough numbers to be suspected of being a disruptive competitor in some patches of *Calamintha ashei* is the European Honey Bee, *Apis mellifera* Linnaeus.

The known distribution and natural history of *Osmia calaminthae* suggest that it may be among the most geographically restricted and host specific bees in eastern North America. It is possible that *Osmia calaminthae* has wider geographic and host plant ranges than now known, as there has never been a comprehensive attempt to survey the bees of Florida, or to document their hosts. There are, however, biogeographic factors that could explain narrow geographic and host ranges for this species.

*Osmia calaminthae* is known from Florida scrub habitat on the southern half of the Lake Wales Ridge. Florida scrub is a unique shrub community found on ridges and knolls of wind-deposited silica sand. Vegetation consists of small, sclerophyllous oaks and a variety of other shrubs and small trees. The low and open structure of the habitat is maintained by occasional fires. For a more complete description of Florida scrub, see Myers (1990). An important sub-category of Florida scrub habitat is the “scrub rosemary bald.” Scrub Rosemary, *Ceratiola ericoides* Michx. (also known as Sandhill- or Florida-Rosemary, or Sand Heath), is an aberrant member of the Ericaceae that occurs on the most highly drained scrub sites, often forming nearly pure stands of bushes that grow to be about 1.5 m high, with patches of bare sand between the clumps of *Calamintha ericoides* (Johnson 1982). *Calamintha ashei* and several other narrowly distributed Florida scrub specialist plants seem to need bare sand patches in scrub rosemary balds, although some of these plants can move into areas of bare sand caused by human disturbance. Several of the plants found in rosemary balds have host-specific insects, including *Calamintha ashei*, which is the host of the plant bug *Keltonia clinopodii* Kelton (Miridae) (Kelton 1966). Palynological evidence shows that *Ceratiola* barrens were much more widespread in south Florida at various times during the last 40,000 years (Delcourt and Delcourt 1981, Trapnell et al. 2007).

The Lake Wales Ridge, especially its southern half where *Osmia calaminthae* occurs, is the area with the largest number of Florida scrub specialist arthropods and plants, and has the only concentration of scrub specialists not found elsewhere in Florida (Deyrup 1990). This is probably due to the large size of the Lake Wales Ridge, its relative antiquity (over one million years), its southern position, and its uneven topography, which may affect fire frequency (Deyrup 1990). About 90 species of Florida scrub specialist arthropods are known from the Lake Wales Ridge, although many of these also occur in scrub areas elsewhere in Florida (Deyrup 2011, unpublished list). *Calamintha ashei* is largely restricted to the Lake Wales Ridge (Turner et al. 2006). Another spring-blooming woody mint, *Conradina brevifolia* Shinners, has a similar floral structure, although it is yet to be associated with *Osmia calaminthae*. *Conradina brevifolia* is largely restricted to a small area on the Lake Wales Ridge (Turner et al. 2006).

*Osmia calaminthae* is currently known from only four sites within an area about 20 km long and 2 km wide on the Lake Wales Ridge. Archbold Biological Station is a private research station on the southern end of the Lake Wales Ridge where natural habitats have been protected since 1941. Two other protected sites are Lake Wales Ridge Wildlife and Environmental Areas managed by the Florida Fish and Wildlife Conservation Commission. One of these areas, Gould Road Preserve, has a large population of *Calamintha ashei* and apparently a substantial population of *Osmia calaminthae*, but the site is unfenced and subject to pesticide drift from adjacent orange groves. The other protected site, Holmes Avenue Preserve, also has a large population of *Calamintha ashei*, but is similarly unfenced, includes a large number of small, unacquired private parcels, and is subject to destructive use by off-road vehicles. There are over 20 Florida scrub preserves on the Lake Wales Ridge, but many of these lack large populations of *Calamintha ashei*. The majority of *Osmia calaminthae* records are from scattered undeveloped lots in Placid Lakes, a platted subdivision south of the town of Lake Placid; this collection area is unprotected.

The nest site of *Osmia calaminthae* is unknown, but nest sites are less likely to be a limiting factor for this species than a scarcity of floral hosts and habitat. If nests are in dead wood, fires that remove most dead wood could affect populations of *Osmia calaminthae*.

Considering all the factors discussed above, attention should be given to the conservation needs of this recently discovered bee. The type locality of this species, where most known individuals have been observed, photographed, and collected, is a site with many vacant lots within a subdivision. Future full residential development at this site would threaten much of the known habitat of this species. *Osmia calaminthae*, remains, however, an extremely poorly known insect. It has a short flight period, and is therefore easily overlooked. It could be more abundant and widespread than it appears at present. Surveys of additional potential habitat, i.e., areas of sand scrub where the host plant occurs, are urgently needed to better assess whether the few known sites are critical habitat for the species or if it is more widely distributed but under recorded.

### Osmia conjunctoides

Unfortunately, *Osmia conjunctoides* has been too rarely collected to allow for more than a few speculative comments on its conservation status; none of the authors have seen this bee in life. Only one examined specimen, the holotype of *Osmia subfasciata miamiensis*, has a recorded host plant associated with it (*Crotalaria pumila* Ortega); thus, it is not possible to say if the species is restricted in its plant host use. However, the presence of nearly uniformly short, erect hairs on the face of females is rare among *Osmia* in North America; in fact, this particular facial hair morphology is only known from *Osmia conjunctoides* and *Osmia calaminthae*. It is therefore reasonable to suspect that *Osmia conjunctoides* is associated with a restricted set of floral host species or with a particular floral morphology. At least six other *Osmia* from North America have facial hair modifications suggestive of pollen collection from nototribic flowers (V. H. Gonzalez, Griswold, & Rightmyer, unpublished data); the hairs on these bees either form a basket of stiff, proclinate hairs on the vertex and frons, or are cork-screw shaped on the clypeus and frons. Although the hair morphology is not identical, the placement of the modified hairs on the clypeus and frons appears to be associated with pollen collection from nototribic flowers in both Old and New World *Osmia*, usually from the family Lamiaceae (although polylecty with preference for both Lamiaceae and Fabaceae has been documented; Müller 1996) or Scrophulariaceae.

A strong association of *Osmia conjunctoides* with *Crotalaria pumila* is tenuous. This legume has anthers that are enclosed in a narrow passageway in the keel, and it is not yet known if a bee collecting pollen or nectar would contact the anthers of this plant with her head. However, in alfalfa (*Medicago sativa* Linnaeus), another plant in the family Fabaceae (albeit an unusual one), the act of pollination changes the placement of the anthers from a ventral position to a more dorsal one (i.e., “tripping” the flower, causing the staminal column to snap upward toward the banner petal; Pitts-Singer and Cane 2011). Indeed, for *Medicago sativa*, *Megachile rotundata* (Fabricius) is preferred over honey bees for managed pollination due to its tolerance for being struck repeatedly on the head by the anthers of this flower.  Thus, although *Crotolaria* is not likely the main host plant of *Osmia conjunctoide*s, it cannot be ruled out until field observations have taken place. It is also unknown if the distribution of *Crotolaria pumila* has any predictive value for the distribution of *Osmia conjunctoides*. However, as it is the only floral information available to us for *Osmia conjunctoides* we provide a few comments on the natural history of this plant species and a related species within the same genus. *Crotalaria pumila* occurs in beach dunes and coastal pinelands along the Atlantic Coast from Brevard County south (Taylor 1998). Although much of this range has been heavily developed or overrun by exotic plants, patches of native habitat can be found in a series of coastal preserves, the largest of which are Jonathan Dickinson State Park, Hobe Sound National Wildlife Preserve, and Merritt Island National Wildlife Preserve. Cape Florida, where the holotype of *Osmia subfasciata miamiensis* was collected, might still have a protected population of this species within Bill Baggs Cape Florida State Park. St. Catherines Island is a National Historic Landmark and is also a protected area. *Crotalaria rotundifolia* Walter ex J. F. Gmel. might also be a potential plant to investigate when searching for further specimens of *Osmia conjunctoides*; *Crotolaria rotundifolia* is more widespread in Florida, and inland populations are dependent upon fire. The most recently collected specimens were from a burn zone in Seminole County. Thus, open, frequently burned or disturbed areas (i.e., Brooksville Ridge in Citrus and Levy Counties) are potential habitat.

Until further individuals of *Osmia conjunctoides* have been located and studied, the conservation status of this species will remain unclear. It is potentially an endangered species, considering the massive scale of destruction and alteration of most of its presumed coastal habitat. The decrease of fire frequency in many inland areas of the southeastern United States may also be a factor in this species’ conservation status, although the species has yet to be associated with a fire-dependent plant.

## Supplementary Material

XML Treatment for Osmia (Melanosmia) calaminthae

XML Treatment for Osmia (Diceratosmia) conjunctoides
